# Effects of CYP2C19 genetic polymorphisms on the cure rates of *H. pylori* in patients treated with the proton pump inhibitors: An updated meta-analysis

**DOI:** 10.3389/fphar.2022.938419

**Published:** 2022-10-06

**Authors:** Xianghong Zhao, Zhongqiu Zhang, Fang Lu, Mengqiu Xiong, Liping Jiang, Ke Tang, Min Fu, Yu Wu, Bangshun He

**Affiliations:** ^1^ Department of Clinical Pharmacy, School of Basic Medicine and Clinical Pharmacy, China Pharmaceutical University, Nanjing, China; ^2^ Division of Clinical Pharmacology, General Clinical Research Center, Nanjing First Hospital, Nanjing Medical University, Nanjing, China; ^3^ Department of Pharmacy, Nanjing First Hospital, China Pharmaceutical University, Nanjing, China; ^4^ Department of Laboratory Medicine, Nanjing First Hospital, Nanjing Medical University, Nanjing, China; ^5^ H. pylori Research Key Laboratory, Nanjing Medical University, Nanjing, China

**Keywords:** *Helicobacter pylori*, eradication rate, genetic polymorphisms, CYP2C19, meta- analysis

## Abstract

**Background:** The cure rates of *Helicobacter pylori* (*H. pylori*) treatment using a proton pump inhibitor (PPI) are gradually decreasing due to antibiotic resistance, poor compliance, high gastric acidity, and cytochrome P450 2C19 (CYP2C19) polymorphism, and the effects of PPI depend on metabolic enzymes, cytochrome P450 enzymes. The aim of this meta-analysis was to determine whether CYP2C19 polymorphisms affect *H. pylori* cure rates in patients treated with different proton pump inhibitors (PPIs) according to stratified analysis.

**Materials and methods:** The literature was searched with the key words “*H. pylori*” and “CYP2C19” in PubMed, CNKI, and Wanfang up to 31 May 2022, and the studies were limited to clinical observational or randomized controlled trials (RCTs). Finally, seven RCTs and 29 clinical observational studies met the inclusion criteria and were used for the meta-analysis *via* STATA version 16.

**Results:** The cure rates were significantly different between genotypes of homozygous extensive metabolizers (EM) and poor metabolizers (PM) (OR = 0.58, 95% CI: 0.47–0.71) and between EM and heterozygous extensive metabolizers (IM) (OR = 0.71, 95% CI: 0.59–0.86), but not between IM and PM. Moreover, there was a significantly lower *H. pylori* cure rate in EM subjects than that in IM subjects when treated with omeprazole (66.4% vs. 84.1%), lansoprazole (76.1% vs. 85.6%), but not rabeprazole, esomeprazole, or pantoprazole. In addition, there was a significantly lower *H. pylori* cure rate in EM subjects than that in IM subjects when treated with a PPIs for 7 days (77.4% vs. 82.1%), but not 14 days (85.4% vs. 90.0%).

**Conclusion:** Carriers of CYP2C19 loss-of-function variant alleles (IM and PM) exhibit a significantly greater cure rate of *H. pylori* than noncarriers (EM) regardless of other factors (84.7% vs. 79.2%). In addition, pantoprazole- and rabeprazole-based quadruple therapy for *H. pylori* treatment is less dependent on the CYP2C19 genotype and should be prioritized in Asian populations with *H. pylori*.

## Introduction

*Helicobacter pylori* (*H. pylori*) infection is a major risk factor for peptic ulcer and gastritis and is also associated with mucosal-associated lymphoid tissue (MALT) lymphoma and gastric cancer (Marshall and Warren, 1984; Yamada, 1994; Marshall and Windsor, 2005; Fischbach and Malfertheiner, 2018). According to the content of the Kyoto global consensus, the *H. pylori* infection exceeded 50% of the general populations worldwide, and *H. pylori* control has become an important issue for the Centers for Disease Control and Prevention in the world (Sugano et al., 2015).

At present, triple therapy and quadruple therapies are mainly used for *H. pylori* treatment all over the world due to the high cure rate; however, large-scale *H. pylori* treatments have resulted in increasing rates of resistance to multiple antibiotics, together with factors such as drug compliance, inappropriate treatment regimens, therapy duration, intragastric acidity, and CYP2C19 genetic polymorphisms, resulting in a gradual decline in *H. pylori* cure rates (Zhong et al., 2022). Moreover, previous studies indicated that *H. pylori* cure rates may vary by the use of different proton pump inhibitors (PPIs), whose effects are affected by genetic polymorphisms of drug-metabolizing enzymes CYP2C19 (Chaudhry et al., 2008; Klotz, 2009).

The fields of medicine where clinical outcomes are particularly dependent on CYP2C19 polymorphisms are gastroenterology, cardiology, psychiatry, fungology, and oncology. CYP2C19 is involved in the metabolism of PPIs and therefore it can influence reflux therapy, ulcer prevention, and *H. pylori* therapy. The CYP2C19 enzyme also plays an important role in two bioactivation steps of clopidogrel, leading to a lower (CYP2C19*17 carriers) or higher (CYP2C19*2 carriers) risk of major adverse cardiovascular events. It affects antidepressant therapy and methadone replacement therapy as well as voriconazole prophylaxis. Moreover, in breast cancer patients treated with tamoxifen, the presence of the *2 allele was associated with a longer recurrence-free time or better survival, while the *17 allele was associated with a more favorable outcome (Sienkiewicz-Oleszkiewicz et al., 2018). Our study focused on the effect of the CYP2C19 genotype on the metabolism of PPIs and consequently on the cure rate of *H. pylori*.

CYP2C19 is a major drug-metabolizing enzyme for the clearance of the first-generation PPIs omeprazole and lansoprazole (∼80%) with a relatively lesser contribution of CYP3A4. In contrast, the second-generation PPIs esomeprazole and rabeprazole are less dependent on CYP2C19 in their metabolism, suggesting that they may be less influenced by genetic variability in CYP2C19 compared to the first-generation PPIs (Lima et al., 2021).

**FIGURE 1:**
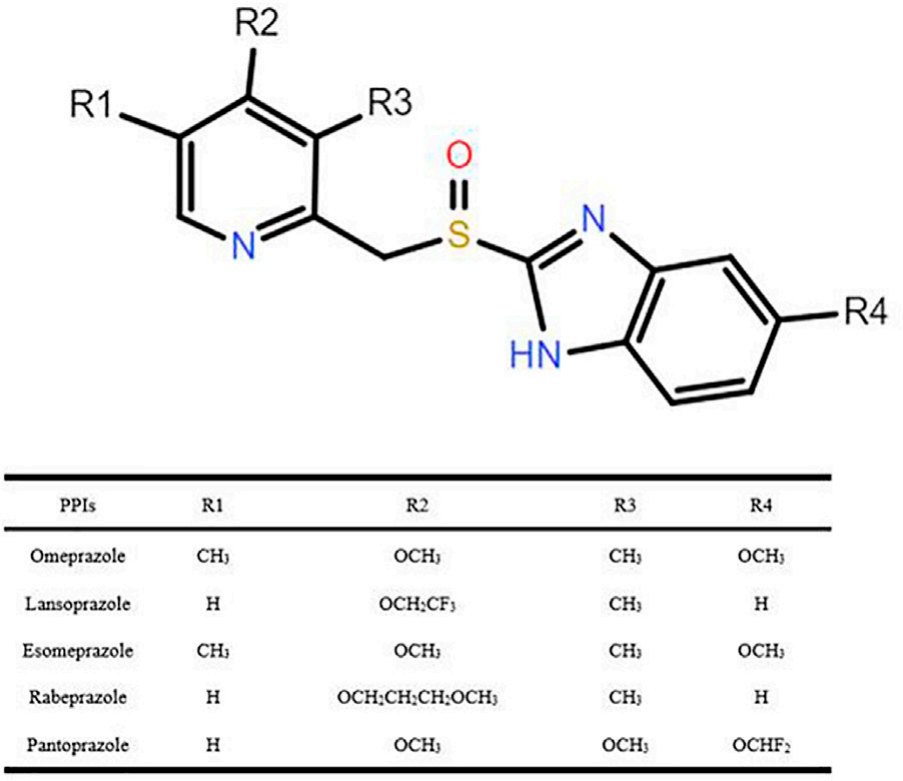
Structures of proton pump inhibitors discussed.

The PPIs, a class of drugs that suppress gastric acid production through irreversible inhibition of the H^+^/K^+^-ATPase (or proton pump) (Goldstein et al., 2017), have been used to treat gastric acid–associated disorders, such as gastroesophageal reflux and peptic ulcer. Six PPIs are currently approved in the United States including omeprazole, the prototype in this class, lansoprazole, dexlansoprazole, pantoprazole, rabeprazole, and esomeprazole (a stereoisomer of omeprazole) (El Rouby et al., 2018), and five of these reagents were explored in our study (i.e., omeprazole, lansoprazole, pantoprazole, rabeprazole, and esomeprazole), as shown in Figure 1. PPI metabolism has been studied in adults, and thus the PK parameters summarized in Table 1 (Li et al., 2004; Shin and Kim, 2013; Ward and Kearns, 2013; Yu et al., 2017; Savarino et al., 2018) apply to adults. There are some differences in the extent to which PPIs are metabolized by CYP2C19, leading to variability in their PK and pharmacodynamic (PD) parameters, ultimately impacting their efficacy. It is documented that CYP2C19 is responsible for > 80% of the metabolism of omeprazole, lansoprazole, and pantoprazole metabolism (Andersson et al., 1998). However, previous studies have confirmed that esomeprazole is metabolized, to less content, by CYP2C19 than omeprazole, resulting in less interindividual variation in plasma drug concentrations than omeprazole (Dent, 2003). Rabeprazole is metabolized to thioether-rabeprazole mainly *via* a non-enzymatic pathway, with minor involvement of CYP2C19 (Tybring et al., 1997).

**TABLE 1:** Pharmacokinetic properties of proton pump inhibitors.

PPI	Hepatic metabolism	Bioavailability (%)	Time to plasma peak level (tmax)	Half-life (T1/2)	Fraction of CYP2C19 metabolism	Pharmacokinetics
Omeprazole	CYP2C19 (major), CYP3A4 (minor)	30%–40%	0.5–3.5 h	0.5–1 h	>80%	Nonlinear
Lansoprazole	CYP2C19 (major), CYP3A4 (minor)	80%–85%	1.7 h	1.6 h	>80%	Linear
Esomeprazole	CYP2C19 (major), CYP3A4 (minor)	64%–90%	1.5 h	1.3–1.6 h	∼70%	Nonlinear
Rabeprazole	Non-enzymatic clearance, minor metabolism through CYP2C19	52%	2–5 h	1–2 h	Minimal	Linear
Pantoprazole	Non-enzymatic clearance, minor metabolism through CYP2C19	77%	2–3 h	1–1.9 h	>80%	Linear

Polymorphic CYP2C19 phenotypes could affect the cure rate of *H. pylori* because some of the PPIs are metabolized predominantly by CYP2C19, whose alleles are categorized into three groups as follows: wild-type function (e.g., CYP2C19*1), loss-of-function (e.g., CYP2C19*2 and *3), and enhanced function (e.g., CYP2C19*17) (Lima et al., 2021). Several phenotypes have been identified for CYP2C19. The extensive or normal metabolizer (EM) is carrier of the *1/*1 or *1/*17 genotypes; the intermediate metabolizer (IM) is carrier of *1/*2, *1/*3, or *2/*17 genotypes; the poor metabolizer (PM) is carrier of *2/*2, *2/*3, or *3/*3 genotypes; and the ultra-rapid metabolizer (UM) is an allele *17 homozygous carrier (*17/*17). EM and UM metabolize PPIs at a fast rate, and thus higher doses of these agents are required in EM and UM to achieve the same effect as that required in IM and PM (Arenas et al., 2019). It is important to note that most CYP2C19 studies evaluating PPIs were conducted in Asian populations, in whom the allele frequency of the CYP2C19*17 is lower than that in non-Asian populations, therefore, few studies about CYP2C19 EMs and UMs have been published to date (Lima et al., 2021). Following administration of standard doses of the first-generation PPIs, CYP2C19 IM and PM experienced higher PPI AUC (3–14 fold) and C_max_ (2–6 fold) than CYP2C19 EM as a result of reduced PPI clearance *via* the CYP2C19 pathway (Chang et al., 1995; He et al., 2003; Qiao et al., 2006). The increased PPI exposure in CYP2C19 IM and PM has been linked to improved acid suppression (i.e., higher intragastric pH and longer time with pH > 4.0) and improved therapeutic benefits. Thus, CYP2C19 IM and PM are considered to be “therapeutically advantaged” compared to EM in terms of efficacy (Furuta et al., 1999; Shimatani et al., 2003; Kurzawski et al., 2006; Park et al., 2017).

In clinical practice, EMs produce an abundance of more active enzymes and metabolize the PPIs at a higher rate, limiting the drugs’ bioavailability and consequently lowering their antisecretory efficacies. IMs contain one wild-type allele and one mutant allele, resulting in the compromised production of the enzyme and thus slower metabolism of the PPI. In the PMs, both alleles are mutated (loss-of-function variant alleles), resulting in a much slower rate of PPI metabolism, ensuring greater bioavailability and subsequently increased antisecretory efficacy. Meanwhile, the frequency of the genotype status is highly varied among different regions (Zhao et al., 2008), which may affect the *H. pylori* treatment. Therefore, the effects of CYP2C19 genotypes on the *H. pylori* cure rates were reported extensively from different treatment regimens, among different geographic regions or ethnic populations.

There are also some factors that we overlook that can affect our results, such as the dose of PPI, antibiotic resistance, and the interleukin (IL)-1β genotype can have an impact on the cure rate of *H. pylori*. It has been reported in the literature that the effect of CYP2C19 genetic polymorphisms on the cure rate of *H. pylori* can be attenuated by increasing the dose of PPIs (omeprazole and lansoprazole) (Padol et al., 2006; Zhao et al., 2008; Tang et al., 2013). The cure rate of *H. pylori* also decreases significantly when metronidazole and clarithromycin resistance is observed (Houben et al., 1999; Miwa et al., 2001). IL-1β can affect the gastric acid secretion and thus mediate the cure of *H. pylori*, but the influence of the IL-1β genotype on treatment of *H. pylori* remains highly controversial (Take et al., 2003; Furuta et al., 2004).

In our study, we investigated the effects of CYP2C19 genetic polymorphisms on *H. pylori* treatment regardless of PPI doses, resistance to antibiotics, medication compliance, and IL-1β genotype and performed subgroup analysis to investigate whether the effects of PPI, treatment duration, treatment regimen, and geographic factors on *H. pylori* treatment were associated with the CYP2C19 genotype. Moreover, we indicated that the main underlying mechanism of *H. pylori* treatment failure is insufficiently sustained gastric acid suppression.

Therefore, the aim of our study was to provide an alternate strategy by which optimizing *H. pylori* treatment would use first-line treatments that show less CYP2C19 genetic polymorphism dependence on cure rates.

## Materials and methods

### Literature search

A computerized systematic literature search was conducted *via* PubMed, China National Knowledge Infrastructure (CNKI), and Wanfang, and the relevant literature was searched up to 31 May 2022 by using the key words “cytochrome P450 2C19” or “CYP2C19,” and “*Helicobacter pylori*” or “*H. pylori*,” Published English review scientific studies were included. At the same time, supplementary retrieval was carried out by browsing references included in the publications, and limited clinical trials and randomized controlled trials (RCTs) were included.

### Inclusion criteria

For this meta-analysis, all searched literature followed the following inclusion criteria: 1) double, triple, and quadruple therapies for 7–14 days; 2) patients positive for *H. pylori* infection prior to treatment; 3) established genotypes of CYP2C19, such as EM, IM, and PM, using a standard method (i.e., polymerase chain reaction-restriction fragment length polymorphism and Taqman probe); 4) full-text articles written in English. The following two parameters were also considered: randomization and blindness (single or double blindness either to treatment or genotype groups). In order to improve the power of the meta-analysis, dropouts/withdrawals were not recorded.

### Data extraction

Two evaluators (XH.Z and ZQ.Z) independently screened the literature according to the inclusion criteria (see aforementioned), extracted the data, and cross-checked them, and any discrepancies were resolved through discussion with a third reviewer (L.F) or referring back to the original articles until the two reviewers reached a consensus. The data of enrolled studies were extracted, including the first author, publication year, study group, a total number of cases, country, age, gender, basic characteristics of patients, treatment regimens, types of PPIs, therapy length, gene detection method, number of expected phenotypic cases of CYP2C19, and the cure rate of *H. pylori*.

### Statistical analysis

After the pooled comparison, cure rates were calculated with the number needed to treat (NNT) and odds ratio (OR) with their corresponding 95% CI of each study by using STATA 16.0 software. For those studies (Sapone et al., 2003; Sugimoto et al., 2007; Miehlke et al., 2008; Burhan et al., 2010; Gawrońska-Szklarz et al., 2010; Jinda et al., 2011; Prasertpetmanee et al., 2013; Chanagune et al., 2014; Sugimoto et al., 2014; Kittichet et al., 2016; Yun-An et al., 2017; Piyakorn et al., 2019; Ke et al., 2021; Yang et al., 2021) with value 0 appeared in the four-grid table, we added 1 to replace 0 for analysis after reading the literature and discussion. Cochrane’s Q-test and *I* ^2^ test were performed to evaluate heterogeneity of enrolled studies (Hoaglin, 2016). If the Cochran’s Q-test probability was 0.05, this means that the study was significantly heterogeneous, and the *I* ^2^ test was used to classify heterogeneity as low (≤25%), medium (≈50%), or high (≥75%) (McNicholl et al., 2012). *p* < 0.05 means the difference was statistically significant. If there was a significant heterogeneity (*p* < 0.05), we selected a random-effects model to pool all the eligible data, otherwise, a fixed-effects model was used. Sensitivity analysis was conducted to examine the stability of the pooled results. Publication bias was assessed using the funnel plot with the Egger’s and Begg’s tests.

## Results

### Characteristics of included studies

**FIGURE 2:**
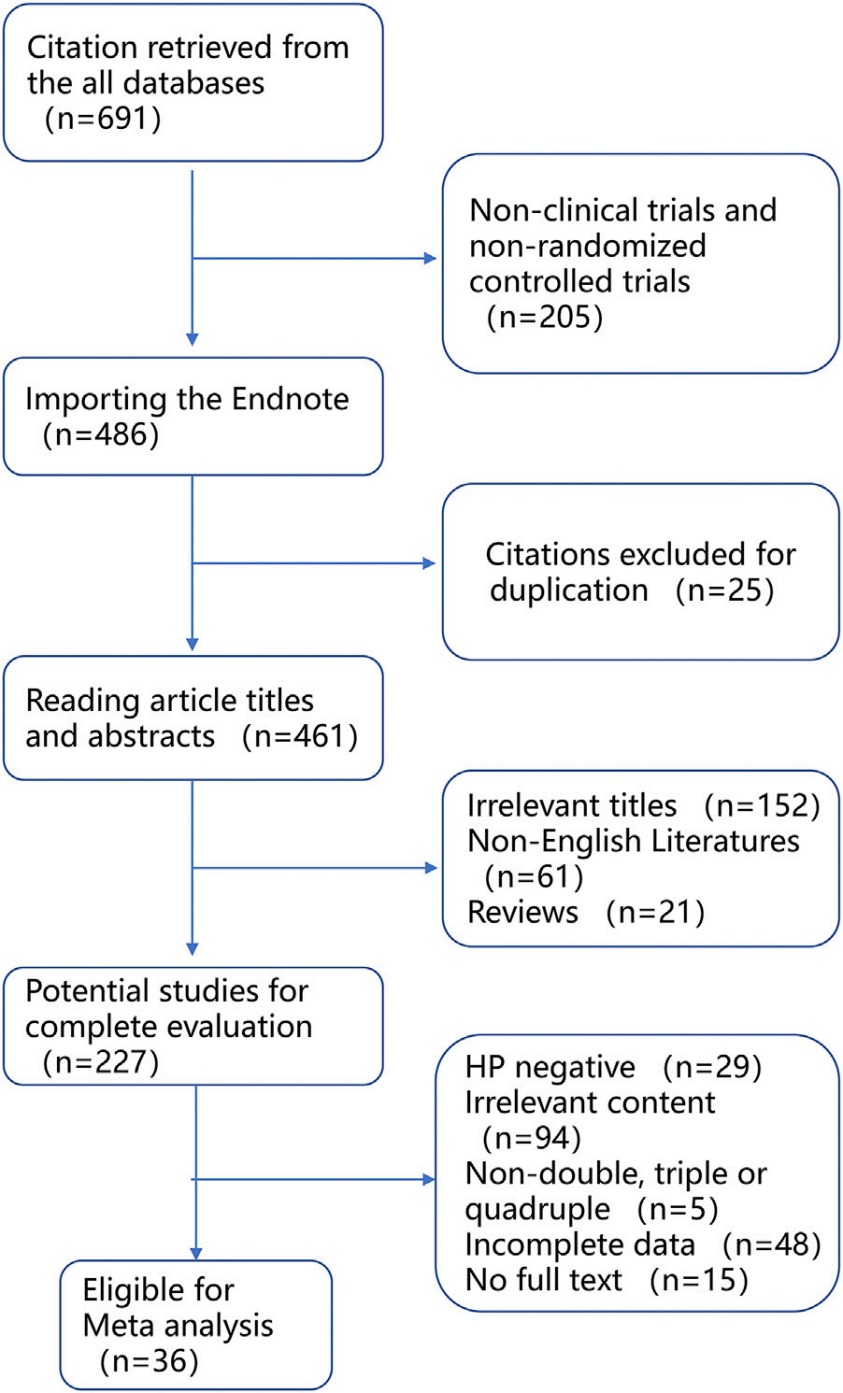
Meta-analysis flow chart.

Overall, a total of 36 articles (Dojo et al., 2001; Inaba et al., 2002; Bae et al., 2003; Kawabata et al., 2003; Miki et al., 2003; Sapone et al., 2003; Take et al., 2003; Suzuki et al., 2007; Sheu et al., 2005; Ishida et al., 2006; Furuta et al., 2007; Kuwayama et al., 2007; Sugimoto et al., 2007; Miehlke et al., 2008; Jung-Hwan et al., 2009; Burhan et al., 2010; Gawrońska-Szklarz et al., 2010; Hoon et al., 2010; Lee et al., 2010; Pan et al., 2010; Shyan et al., 2010; Zhang et al., 2010; Jinda et al., 2011; Prasertpetmanee et al., 2013; Ahmad et al., 2014; Chanagune et al., 2014; Lee et al., 2014; Sugimoto et al., 2014; Kittichet et al., 2016; Yun-An et al., 2017; Piyakorn et al., 2019; Woon et al., 2019; Song et al., 2020; Sugimoto et al., 2020; Ke et al., 2021; Yang et al., 2021) were included in the meta-analysis from 691 relevant reports according to the inclusion criteria (Figure 2; Table 2). To conduct subgroup analysis, studies (Dojo et al., 2001; Inaba et al., 2002; Bae et al., 2003; Kawabata et al., 2003; Miki et al., 2003; Sapone et al., 2003; Take et al., 2003; Furuta et al., 2005; Sheu et al., 2005; Ishida et al., 2006; Furuta et al., 2007; Kuwayama et al., 2007; Sugimoto et al., 2007; Miehlke et al., 2008; Jung-Hwan et al., 2009; Burhan et al., 2010; Gawrońska-Szklarz et al., 2010; Hoon et al., 2010; Lee et al., 2010; Pan et al., 2010; Shyan et al., 2010; Zhang et al., 2010; Jinda et al., 2011; Prasertpetmanee et al., 2013; Ahmad et al., 2014; Chanagune et al., 2014; Lee et al., 2014; Sugimoto et al., 2014; Kittichet et al., 2016; Yun-An et al., 2017; Piyakorn et al., 2019; Woon et al., 2019; Song et al., 2020; Sugimoto et al., 2020; Ke et al., 2021; Yang et al., 2021) were divided into more than one group according to stratified variation, including the type of PPIs, treatment duration, regions, and treatment regimens. In the analysis of the PPI treatment subgroup, a total of 36 research articles were enrolled, which comprised the treatment of omeprazole (Dojo et al., 2001; Inaba et al., 2002; Sapone et al., 2003; Sheu et al., 2005; Zhang et al., 2010; Yun-An et al., 2017), lansoprazole (Inaba et al., 2002; Kawabata et al., 2003; Miki et al., 2003; Furuta et al., 2005; Ishida et al., 2006; Sugimoto et al., 2007; Burhan et al., 2010; Hoon et al., 2010; Prasertpetmanee et al., 2013; Chanagune et al., 2014), esomeprazole (Sheu et al., 2005; Miehlke et al., 2008; Pan et al., 2010; Lee et al., 2014; Song et al., 2020; Ke et al., 2021), rabeprazole (Dojo et al., 2001; Inaba et al., 2002; Bae et al., 2003; Kawabata et al., 2003; Miki et al., 2003; Kuwayama et al., 2007; Hoon et al., 2010; Shyan et al., 2010; Zhang et al., 2010; Sugimoto et al., 2014; Kittichet et al., 2016; Yun-An et al., 2017), and pantoprazole (Jung-Hwan et al., 2009; Gawrońska-Szklarz et al., 2010; Lee et al., 2014). One study reported the use of the PPI vonoprazan (Sugimoto et al., 2020), but meta-analysis could not be performed on a single report, which was not included in the analysis. In the subgroup analysis of the treatment duration, a total of 36 studies (Dojo et al., 2001; Inaba et al., 2002; Bae et al., 2003; Kawabata et al., 2003; Miki et al., 2003; Sapone et al., 2003; Take et al., 2003; Furuta et al., 2005; Sheu et al., 2005; Ishida et al., 2006; Furuta et al., 2007; Kuwayama et al., 2007; Sugimoto et al., 2007; Miehlke et al., 2008; Jung-Hwan et al., 2009; Burhan et al., 2010; Gawrońska-Szklarz et al., 2010; Hoon et al., 2010; Lee et al., 2010; Pan et al., 2010; Shyan et al., 2010; Zhang et al., 2010; Jinda et al., 2011; Prasertpetmanee et al., 2013; Ahmad et al., 2014; Chanagune et al., 2014; Lee et al., 2014; Sugimoto et al., 2014; Kittichet et al., 2016; Yun-An et al., 2017; Piyakorn et al., 2019; Woon et al., 2019; Song et al., 2020; Sugimoto et al., 2020; Ke et al., 2021; Yang et al., 2021) were chosen, including 27 studies (Dojo et al., 2001; Inaba et al., 2002; Bae et al., 2003; Kawabata et al., 2003; Sapone et al., 2003; Take et al., 2003; Furuta et al., 2005; Sheu et al., 2005; Ishida et al., 2006; Furuta et al., 2007; Kuwayama et al., 2007; Sugimoto et al., 2007; Miehlke et al., 2008; Jung-Hwan et al., 2009; Gawrońska-Szklarz et al., 2010; Hoon et al., 2010; Lee et al., 2010; Pan et al., 2010; Zhang et al., 2010; Prasertpetmanee et al., 2013; Chanagune et al., 2014; Lee et al., 2014; Piyakorn et al., 2019; Woon et al., 2019; Sugimoto et al., 2020) exerting 7-day treatment, and 10 studies (Burhan et al., 2010; Shyan et al., 2010; Prasertpetmanee et al., 2013; Ahmad et al., 2014; Chanagune et al., 2014; Kittichet et al., 2016; Piyakorn et al., 2019; Song et al., 2020; Ke et al., 2021; Yang et al., 2021) reporting 14-day treatment, respectively. In the subgroup analysis of the region, a total of 35 articles were included, the majority of studies were from Asia (13 in Japan, nine in China, five in South Korea, four in Thailand, and one in Turkey), followed by Europe (one in Germany, Poland, and Italy each). In subgroup analysis of regimens, including 31 studies (Dojo et al., 2001; Inaba et al., 2002; Bae et al., 2003; Kawabata et al., 2003; Miki et al., 2003; Sapone et al., 2003; Take et al., 2003; Furuta et al., 2005; Sheu et al., 2005; Ishida et al., 2006; Furuta et al., 2007; Kuwayama et al., 2007; Sugimoto et al., 2007; Miehlke et al., 2008; Jung-Hwan et al., 2009; Burhan et al., 2010; Gawrońska-Szklarz et al., 2010; Hoon et al., 2010; Lee et al., 2010; Pan et al., 2010; Shyan et al., 2010; Zhang et al., 2010; Jinda et al., 2011; Prasertpetmanee et al., 2013; Ahmad et al., 2014; Lee et al., 2014; Sugimoto et al., 2014; Kittichet et al., 2016; Yun-An et al., 2017; Woon et al., 2019; Song et al., 2020) about triple therapy, five studies (Chanagune et al., 2014; Piyakorn et al., 2019; Sugimoto et al., 2020; Ke et al., 2021; Yang et al., 2021) about quadruple therapy, and only one study (Ke et al., 2021) about double therapy met the inclusion criteria, but no analysis could be performed on a single study and it was excluded.

**TABLE 2:** Summary of articles included in the meta-analysis.

Reference	N	Country	Age	Gender		Basic characteristics of patient	Treatment regimen	PPI	Length (days)	Gene detection method	Type of CYP2C19 (n/N)		
				Male	Female						EM	IM	PM
Zhang et al. (2010)	240	China	45.2 ± 14.2	194	46	GU and DU	Triple therapy	Rabeprazole or omeprazole	7	NA	61/74	99/124	38/44
Zhang et al. (2010)	240	China	45.2 ± 14.2	194	46	GU and DU	Triple therapy	Omeprazole	7	NA	28/39	49/61	18/20
Zhang et al. (2010)	240	China	45.2 ± 14.2	194	46	GU and DU	Triple therapy	Rabeprazole	7	NA	33/35	50/63	20/22
Sheu et al. (2005)	200	China	41.7	99	101	Dyspepsia	Triple therapy	Omeprazole or esomeprazole	7	NA	70/91	55/65	40/43
Sheu et al. (2005)	200	China	41.7	99	101	Dyspepsia	Triple therapy	Omeprazole	7	NA	31/45	27/32	21/23
Sheu et al. (2005)	200	China	41.7	99	101	Dyspepsia	Triple therapy	Esomeprazole	7	NA	39/46	28/33	19/21
Pan et al. (2010)	184	China	44.8	85	99	PUD and gastritis	Triple therapy	Esomeprazole or rabeprazole	7	PCR-RFLP	41/50	50/61	32/36
Pan et al. (2010)	184	China	44.8	85	99	PUD and gastritis	Triple therapy	Esomeprazole	7	PCR-RFLP	25/31	37/40	21/22
Pan et al. (2010)	184	China	44.8	85	99	PUD and gastritis	Triple therapy	Rabeprazole	7	PCR-RFLP	16/19	13/21	11/14
Lee et al. (2010)	204	China	46.8 ± 9.7	68	136	NUD	Triple therapy	Esomeprazole or rabeprazole	7	PCR-RFLP	74/92	67/84	24/28
Woon et al. (2019)	190	Korea	55.4 ± 10.2	89	101	PUD and CG	Triple therapy	NA	7	PCR-RFLP	52/74	52/75	26/41
Lee et al. (2014)	2202	Korea	52.9 ± 12.8	1153	1049	Gastritis, PUD, GD, GC and NUD	Triple therapy	Esomeprazole or omeprazole or lansoprazole or pantoprazole	7	NA	255/325	288/369	99/114
Lee et al. (2014)	2202	Korea	52.9 ± 12.8	1153	1049	Gastritis, PUD, GD, and GC	Triple therapy	Esomeprazole	7	NA	144/184	184/227	65/75
Lee et al. (2014)	2202	Korea	52.9 ± 12.8	1153	1049	Gastritis, PUD, GD, GC and NUD	Triple therapy	Pantoprazole	7	NA	98/119	89/119	30/33
Jung-Hwan et al. (2009)	210	Korea	56.7 ± 10.7	111	99	GU, DU and FD	Triple therapy	Pantoprazole	7	PCR-RFLP	52/60	90/111	32/39
Hoon et al. (2010)	463	Korea	57	276	187	PUD	Triple therapy	Lansoprazole or rabeprazole	7	PCR-RFLP	122/171	168/219	58/73
Hoon et al. (2010)	463	Korea	57	276	187	PUD	Triple therapy	Lansoprazole	7	PCR-RFLP	63/85	87/108	35/41
Hoon et al. (2010)	463	Korea	57	276	187	PUD	Triple therapy	Rabeprazole	7	PCR-RFLP	59/86	81/111	23/32
Bae et al. (2003)	116	Korea	48 ± 13	85	31	PUD	Triple therapy	Rabeprazole	7	PCR-RFLP	42/50	40/46	16/20
Piyakorn et al. (2019)	100	Thailand	54	28	72	NA	Quadruple therapy	NA	7 or 14	NA	22/30	43/50	12/13
Piyakorn et al. (2019)	100	Thailand	54	28	72	NA	Quadruple therapy	NA	7	NA	15/23	18/21	5/6
Piyakorn et al. (2019)	100	Thailand	54	28	72	NA	Quadruple therapy	NA	14	NA	7/7	25/29	7/7
Sapone et al. (2003)	143	Italy	NA	90	53	GU and DU	Triple therapy	Omeprazole	7	PCR-RFLP	70/116	21/25	9/9
Gawrońska-Szklarz et al. (2010)	139	Poland	50.1 ± 14.4	65	74	PUD	Triple therapy	Pantoprazole	7	PCR-RFLP	32/47	53/71	2/2
Miehlke et al. (2008)	103	Germany	52	36	67	NUD and PUD	Triple therapy	Esomeprazole	7	PCR-RFLP	50/66	23/25	4/4
Chanagune et al. (2014)	50	Thailand	NA	17	33	NA	Quadruple therapy	Lansoprazole	7 or 14	NA	39/40	46/47	10/10
Chanagune et al. (2014)	50	Thailand	NA	17	33	NA	Quadruple therapy	Lansoprazole	7	NA	20/21	20/21	7/7
Chanagune et al. (2014)	50	Thailand	NA	17	33	NA	Quadruple therapy	Lansoprazole	14	NA	19/19	26/27	3/3
Take et al. (2003)	249	Japan	48.7 ± 7.2	219	30	PUD	Triple therapy	Omeprazole or lansoprazole or rabeprazole	7	PCR-RFLP	56/81	93/125	36/43
Sugimoto et al. (2014)	153	Japan	56.3 ± 11.0	91	62	NA	Triple therapy	Rabeprazole	7	NA	66/70	59/60	23/23
Miki et al. (2003)	145	Japan	48.9 ± 17.3	113	32	Gastritis and PUD	Triple therapy	Lansoprazole or rabeprazole	7	PCR-RFLP	39/44	64/72	17/22
Miki et al. (2003)	145	Japan	48.9 ± 17.3	113	32	Gastritis and PUD	Triple therapy	Lansoprazole	7	PCR-RFLP	10/12	22/26	7/9
Miki et al. (2003)	145	Japan	48.9 ± 17.3	113	32	Gastritis and PUD	Triple therapy	Rabeprazole	7	PCR-RFLP	29/32	42/46	10/13
Sugimoto et al. (2007)	32	Japan	53.3	20	12	Gastritis, GU, DU and GC	Triple therapy	Lansoprazole	7	PCR-RFLP	7/11	10/13	7/8
Kuwayama et al. (2007)	459	Japan	50.5 ± 12.7	331	128	GU and DU	Triple therapy	Rabeprazole	7	PCR-RFLP	128/149	204/230	77/80
Ishida et al. (2006)	210	Japan	20–69	91	119	NA	Triple therapy	Lansoprazole	7	PCR-CTPP	14/20	31/33	12/14
Inaba et al. (2002)	183	Japan	55	142	41	PUD	Triple therapy	Omeprazole or lansoprazole or rabeprazole	7	PCR-RFLP	49/65	77/87	24/27
Inaba et al. (2002)	183	Japan	55	142	41	PUD	Triple therapy	Omeprazole	7	PCR-RFLP	16/21	24/27	9/10
Inaba et al. (2002)	183	Japan	55	142	41	PUD	Triple therapy	Lansoprazole	7	PCR-RFLP	18/20	26/29	8/9
Inaba et al. (2002)	183	Japan	55	142	41	PUD	Triple therapy	Rabeprazole	7	PCR-RFLP	15/24	27/31	7/8
Kawabata et al. (2003)	187	Japan	52	138	49	PUD	Triple therapy	Rabeprazole or lansoprazole	7	PCR-RFLP	50/63	69/88	16/18
Kawabata et al. (2003)	187	Japan	52	138	49	PUD	Triple therapy	Rabeprazole	7	PCR-RFLP	26/30	43/53	6/10
Kawabata et al. (2003)	187	Japan	52	138	49	PUD	Triple therapy	Lansoprazole	7	PCR-RFLP	24/33	26/35	10/12
Furuta et al. (2005)	141	Japan	51	122	19	PUD and gastritis	Triple therapy	Lansoprazole	7	PCR-RFLP	26/45	60/68	24/26
Sugimoto et al. (2020)	307	Japan	62.3 ± 13.1	160	147	NG and GA	Triple therapy	Vonoprazan	7	NA	70/79	145/170	43/48
Dojo et al. (2001)	170	Japan	43.6 ± 0.6	87	83	CG	Triple therapy	Omeprazole or rabeprazole	7	PCR-RFLP	39/51	65/77	31/36
Dojo et al. (2001)	170	Japan	43.6 ± 0.6	87	83	CG	Triple therapy	Omeprazole	7	PCR-RFLP	22/30	31/36	17/20
Dojo et al. (2001)	170	Japan	43.6 ± 0.6	87	83	CG	Triple therapy	Rabeprazole	7	PCR-RFLP	17/21	34/41	14/16
Burhan et al. (2010)	105	Turkey	46 ± 13.8	42	63	CG	Triple therapy	Lansoprazole	14	PCR-RFLP	54/76	23/24	4/5
Suzuki et al. (2007)	142	Japan	40–69	78	64	PUD	Triple therapy	Lansoprazole	7	NA	7/11	10/13	7/8
Yun-An et al. (2017)	160	China	45.2 ± 12.6	77	83	DS	Triple therapy	Omeprazole or rabeprazole	10	PCR-RFLP	39/54	67/79	33/33
Yun-An et al. (2017)	160	China	45.2 ± 12.6	77	83	DS	Triple therapy	Omeprazole	10	PCR-RFLP	17/28	32/38	12/12
Yun-An et al. (2017)	160	China	45.2 ± 12.6	77	83	DS	Triple therapy	Rabeprazole	10	PCR-RFLP	22/26	35/40	11/11
Shyan et al. (2010)	95	China	46.5 ± 6.0	71	24	Cirrhosis and PUD	Triple therapy	Rabeprazole	14	PCR-RFLP	34/42	34/38	15/15
Song et al. (2020)	380	China	17–70	171	209	NA	Dual therapy or quadruple therapy	Esomeprazole	14	NA	262/301	285/326	90/95
Song et al. (2020)	380	China	17–70	171	209	NA	Dual therapy	Esomeprazole	14	NA	139/155	147/162	45/47
Song et al. (2020)	380	China	17–70	171	209	NA	Quadruple therapy	Esomeprazole	14	NA	123/146	138/164	45/48
Ke et al. (2021)	207	China	39 ± 11.52	115	92	NA	Quadruple therapy	Esomeprazole	14	NA	68/73	82/89	14/14
Kittichet et al. (2016)	50	Thailand	53.6	17	33	NUD	Triple therapy	Rabeprazole	14	NA	23/25	17/21	2/2
Prasertpetmanee et al. (2013)	110	Thailand	51.7	39	71	NA	Triple therapy	Lansoprazole	7 or 14	NA	34/36	19/19	9/9
Prasertpetmanee et al. (2013)	110	Thailand	51.7	39	71	NA	Triple therapy	Lansoprazole	14	NA	19/19	11/11	8/8
Prasertpetmanee et al. (2013)	110	Thailand	51.7	39	71	NA	Triple therapy	Lansoprazole	7	NA	15/17	8/8	1/1
Ahmad et al. (2014)	100	Egyptian	NA	39	61	NA	Triple therapy	Lansoprazole	14	PCR-RFLP	45/65	22/26	7/9
Jinda et al. (2011)	45	Japan	NA	NA	NA	PUD	Triple therapy	NA	NA	SELMAP-PCR	4/12	26/28	5/5
Yang et al. (2021)	244	China	48.5	141	103	CG and PUD	Quadruple therapy	NA	14	NA	86/97	130/141	4/4

Abbreviations: AG, atrophic gastritis; CG, chronic gastritis; DS, dyspeptic symptoms; DU, duodenal ulcer; EM, homozygous extensive metabolizers; GA, gastric atrophy; GC, gastric cancer; GD, gastric dysplasia; GU, gastric ulcer; IM, heterozygous extensive metabolizers; PM, poor metabolizers; PUD, peptic ulcers disease; n/N, number of patients in whom *H. pylori* has been eradicated/total number of patients; N, total number of patients; NA, not available; NUD, non-ulcer dyspepsia; NG, nodular gastritis.

### The efficacy of CYP2C19 polymorphisms on the overall cure rates of *H. pylori*

**FIGURE 3:**
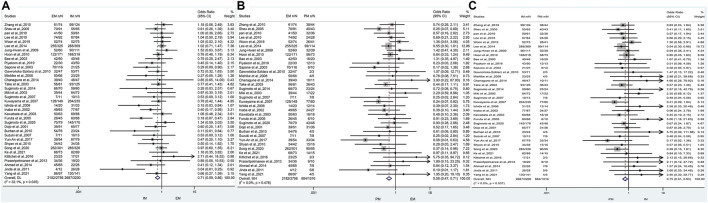
**(A)** Forest plot of EM vs. PM in relation to the *H. pylori* cure rate in the CYP2C19 genotype (*p* = 0.035). **(B)** Forest plot of EM vs. PM in relation to the *H. pylori* cure rate in the CYP2C19 genotype (*p* = 0.478). **(C)** Forest plot of IM vs. PM in relation to the *H. pylori* cure rate in the CYP2C19 genotype (*p* = 0.537). *p* = 0.05, significant heterogeneity, or statistically significant, when appropriate. *p* < 0.05, random-effects model; *p* > 0.05, random-effects model. Symbol: 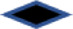, single studies included in the meta-analysis; 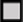, sample size of single studies; 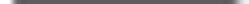, confidence interval (CI); 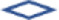, overall pool estimated; 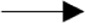, tendency; 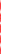, overall pool OR.

The results revealed that, regardless of the type of PPIs, treatment duration regions, and treatment regimens, there was a significant difference in the cure rate of *H. pylori* between EM and IM genotypes (79.2% vs. 84.0%, OR = 0.71, 95% CI: 0.59–0.86, *p* = 0.000, *p*_Heterogeneity_ = 0.035), between EM and PM (79.2% vs. 87.0%, OR = 0.58, 95% CI: 0.47–0.71, *p* = 0.000, *p*_Heterogeneity_ = 0.478), and between IM and PM (84.0% vs. 87%, OR = 0.75, 0.95% CI: 0.61–0.93, *p* = 0.008, *p*_Heterogeneity_ = 0.537) as shown in Figure 3; Table 3.

**TABLE 3:** Efficacy of CYP2C19 polymorphisms on the overall cure rates of *H. pylori*.

Group	EM vs. IM				EM vs. PM				IM vs. PM			
	N	OR (95%CI); sig	Het	Cure rate	N	OR (95%CI); sig	Het	Cure rate	N	OR (95%CI); sig	Het	Cure rate
Overall	5956	0.71 (0.59–0.86), *p* = 0.000	*p* = 0.035	EM (79.2%);	3772	0.58 (0.47–0.71), *p* = 0.000	*p* = 0.478	EM (79.2%);	4216	0.75 (0.61–0.93), *p* = 0.008	*p* = 0.537	IM (84.0%);
				IM (84.0%)				PM (87.0%)				PM (87.0%)

### The efficacy of CYP2C19 polymorphisms on the cure rates of *H. pylori* in the treatment regimens containing different proton pump inhibitors

**FIGURE 4:**
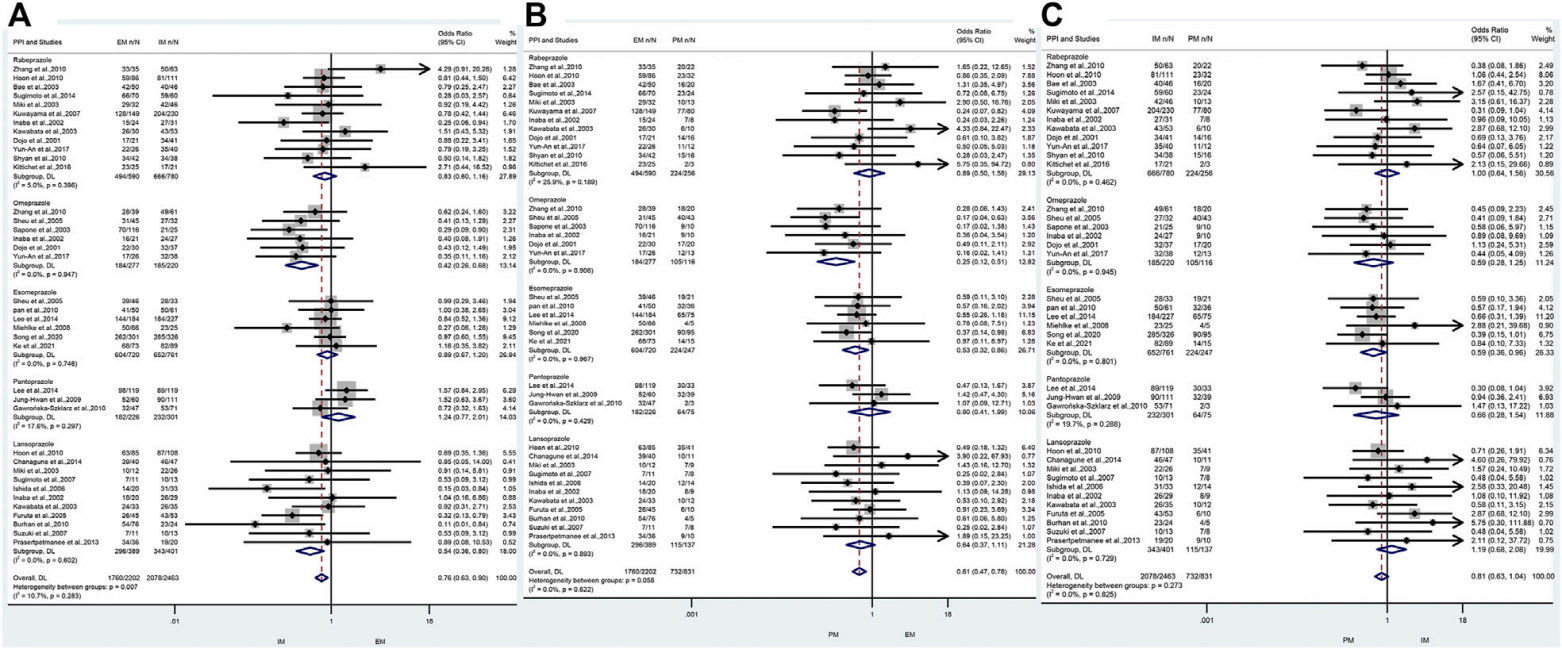
**(A)** Forest plot of EM vs. IM in relation to the *H. pylori* cure rate in the CYP2C19 genotype in different PPIs (*p* = 0.283). **(B)** Forest plot of EM vs. PM in relation to the *H. pylori* cure rate in the CYP2C19 genotype in different PPIs (*p* = 0.622). **(C)** Forest plot of IM vs. PM in relation to the *H. pylori* cure rate in the CYP2C19 genotype in different PPIs (*p* = 0.825). *p* = 0.05, significant heterogeneity or statistically significant, when appropriate. *p* < 0.05, random-effects model; *p* > 0.05, random-effects model. Symbol: 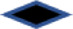, single studies included in the meta-analysis; 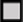, sample size of single studies; 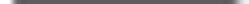, confidence interval (CI); 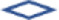, overall pool estimated; 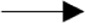, tendency; 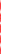, overall pool OR.

In the subgroup of omeprazole-based therapy, there was a significant difference in the *H. pylori* cure rate between EM and IM (66.4% vs. 84.1%, OR = 0.42, 95% CI: 0.26–0.88, *p* = 0.000, *p*_Heterogeneity_ = 0.947) and between EM and PM (66.4% vs. 90.5%, OR = 0.25, 95% CI: 0.12–0.51, *p* = 0.000, *p*_Heterogeneity_ = 0.906), but not between IM and PM (84.1% vs. 90.5%, OR = 0.59, 95% CI: 0.28–1.25, *p* = 0.171, *p*_Heterogeneity_ = 0.945) as shown in Figure 4; Table 4.

**TABLE 4:** Efficacy of CYP2C19 polymorphisms on the cure rates of *H. pylori* in the treatment regimens containing different PPIs.

Subgroup	EM vs. IM				EM vs. PM				IM vs. PM			
	N	OR (95%CI); sig	Het	Cure rate	N	OR (95%CI); sig	Het	Cure rate	N	OR (95%CI); sig	Het	Cure rate
Omeprazole	497	0.42 (0.26–0.88), *p* = 0.000	*p* = 0.947	EM (66.4%);	393	0.25 (0.12–0.51), *p* = 0.000	*p* = 0.906	EM (66.4%);	336	0.59 (0.28–1.25), *p* = 0.171	*p* = 0.945	IM (84.1%);
				IM (84.1%)				PM (90.5%)				PM (90.5%)
Lansoprazole	790	0.54 (0.36–0.80), *p* = 0.002	*p* = 0.602	EM (76.1%);	526	0.64 (0.37–1.11), *p* = 0.111	*p* = 0.893	EM (76.1%);	538	1.19 (0.68–2.08), *p* = 0.542	*p* = 0.729	IM (76.1%);
				IM (85.6%)				PM (83.9%)				PM (83.9%)
Esomeprazole	1481	0.89 (0.67–1.20), *p* = 0.444	*p* = 0.746	EM (83.9%);	967	0.53 (0.32–0.86), *p* = 0.010	*p* = 0.967	EM (83.9%);	1008	0.59 (0.36–0.96), *p* = 0.034	*p* = 0.801	IM (85.7%);
				IM (85.7%)				PM (90.7%)				PM (90.7%)
Rabeprazole	1370	0.83 (0.60–1.16), *p* = 0.272	*p* = 0.396	EM (83.7%);	846	0.89 (0.50–1.58), *p* = 0.699	*p* = 0.189	EM (83.7%);	1036	1.00 (0.64–1.56), *p* = 0.988	*p* = 0.462	IM (85.4%);
				IM (85.4%)				PM (87.5%)				PM (87.5%)
Pantoprazole	527	1.24 (0.77–2.01), *p* = 0.383	*p* = 0.297	EM (80.5%);	301	0.90 (0.41–1.99), *p* = 0.793	*p* = 0.429	EM (80.5%);	376	0.66 (0.28–1.54), *p* = 0.332	*p* = 0.288	IM (76.1%);
				IM (77.1%)				PM (85.3%)				PM (85.3%)

Interestingly, in subgroup analysis of lansoprazole-based therapy, the pooled result showed that there was a significant difference in the *H. pylori* cure rate between EM and IM (76.1% vs. 84.1%, OR = 0.42, 95% CI: 0.36–0.80, *p* = 0.002, *p*_Heterogeneity_ = 0.602), but neither between EM and PM (76.1% vs. 83.9%, OR = 0.64, 95% CI: 0.37–1.11, *p* = 0.111, *p*_Heterogeneity_ = 0.893) nor between IM and PM (76.1% vs. 83.9%, OR = 1.19, 95% CI: 0.68–2.08, *p* = 0.542, *p*_Heterogeneity_ = 0.729) as shown in Figure 3; Table 3. In the subgroup of esomeprazole-based therapy, there was a significant difference in the *H. pylori* cure rate between EM and PM (83.9% vs. 90.7%, OR = 0.53, 95% CI: 0.32–0.86, *p* = 0.010, *p*_Heterogeneity_ = 0.967) and between IM and PM (85.7% vs. 90.7%, OR = 0.59, 95% CI: 0.36–0.96, *p* = 0.034, *p*_Heterogeneity_ = 0.801), but not between EM and IM (83.9% vs. 85.7%, OR = 0.89, 95% CI: 0.67–1.20, *p* = 0.444, *p*_Heterogeneity_ = 0.746) as shown in Figure 4; Table 4.

In contrast, there were no significant differences in the *H. pylori* cure rates between the all genotypes for those patients treated with rabeprazole- or pantoprazole-based regimen as shown in Figure 4; Table 4.

### The efficacy of CYP2C19 polymorphisms on the cure rates of *H. pylori* in 7-day therapy and 14-day therapy

**FIGURE 5:**
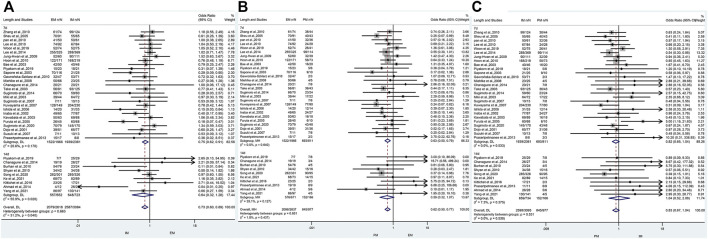
**(A)** Forest plot of EM vs. IM in relation to the *H. pylori* cure rate in the CYP2C19 genotype in different treatment duration (*p* = 0.040). **(B)** Forest plot of EM vs. PM in relation to the *H. pylori* cure rate in the CYP2C19 genotype in different treatment duration (*p* = 0.437). **(C)** Forest plot of IM vs. PM in relation to the *H. pylori* cure rate in the CYP2C19 genotype in different treatment duration (*p* = 0.536). *p* = 0.05, significant heterogeneity or statistically significant, when appropriate. *p* < 0.05, random-effects model; *p* > 0.05, random-effects model. Symbol: 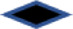, single studies included in the meta-analysis; 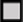, sample size of single studies; 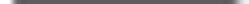, confidence interval (CI); 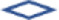, overall pool estimated; 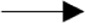, tendency; 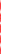, overall pool OR.

Only one study (Yun-An et al., 2017) was just treated for 10 days, but no analysis could be performed on a single study and it was excluded. For those studies with the therapy of 7 days, there was a significant difference in the *H. pylori* cure rate between EM and IM (77.4% vs. 82.1%, OR = 0.75, 95% CI: 0.62–0.91, *p* = 0.004, *p*_Heterogeneity_ = 0.170) and between EM and PM (77.4% vs. 85.5%, OR = 0.63, 95% CI: 0.50–0.79, *p* = 0.000, *p*_Heterogeneity_ = 0.640), but not between IM and PM (82.1% vs. 85.5%, OR = 0.82, 95% CI: 0.65–1.04, *p* = 0.099, *p*_Heterogeneity_ = 0.533). In contrast, when the use of a PPI was maintained for 14 days, there were no significant differences in *H. pylori* cure rates between the three genotypes as shown in Figure 5; Table 5. These results indicated that the CYP2C19 genetic polymorphisms exhibit less important effects on *H. pylori* cure rates in patients treated with a PPI for 2 weeks than those for 1 week in three genotypes.

**TABLE 5:** Efficacy of CYP2C19 polymorphisms on the cure rates of *H. pylori* in 7-day therapy and 14-day therapy.

Subgroup (d)	EM vs. IM				EM vs. PM				IM vs. PM			
	N	OR (95%CI); sig	Het	Cure rate	N	OR (95%CI); sig	Het	Cure rate	N	OR (95%CI); sig	Het	Cure rate
7	4327	0.75 (0.62–0.91), *p* = 0.004	*p* = 0.170	EM (77.4%);	2777	0.63 (0.50–0.79), *p* = 0.000	*p* = 0.640	EM (77.4%);	3177	0.82 (0.65–1.04), *p* = 0.099	*p* = 0.533	IM (82.1%);
				IM (82.1%)				PM (85.5%)				PM (85.5%)
14	1375	0.73 (0.60–0.89), *p* = 0.207	*p* = 0.663	EM (85.4%);	837	0.59 (0.50–0.77), *p* = 0.084	*p* = 0.851	EM (77.4%);	900	1.04 (0.52–2.08), *p* = 0.914	*p* = 0.531	IM (90.0%);
				IM (90.0%)				PM (91.6%)				PM (91.6%)

### The efficacy of CYP2C19 polymorphisms on the cure rates of *H. pylori* in different geographical location of patients

**FIGURE 6:**
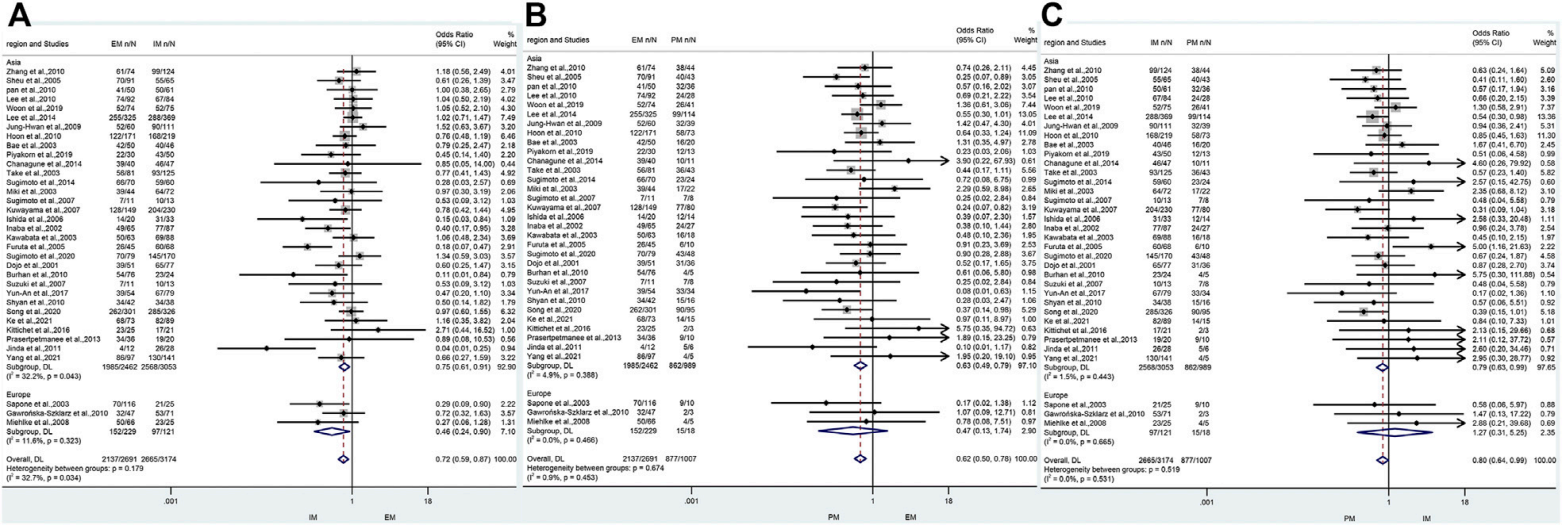
**(A)** Forest plot of EM vs. IM in relation to the *H. pylori* cure rate in the CYP2C19 genotype in different regions (*p* = 0.034). **(B)** Forest plot of EM vs. PM in relation to the *H. pylori* cure rate in the CYP2C19 genotype in different regions (*p* = 0.453). **(C)** Forest plot of IM vs. PM in relation to the *H. pylori* cure rate in the CYP2C19 genotype in different regions (*p* = 0.531). *p* = 0.05, significant heterogeneity, or statistically significant, when appropriate. *p* < 0.05, random-effects model; *p* > 0.05, random-effects model. Symbol: 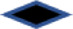, single studies included in the meta-analysis; 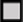, sample size of single studies; 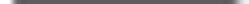, confidence interval (CI); 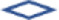, overall pool estimated; 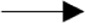, tendency; 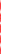, overall pool OR.

In Asia, there was a significant difference in the *H. pylori* cure rate between the all genotypes.

(EM and IM: 80.6% vs. 84.1%, OR = 0.75, 95% CI: 0.61–0.91, *p* = 0.003, P_Heterogeneity_ = 0.043; EM and PM: 80.6% vs. 87.2%, OR = 0.63, 95% CI: 0.49–0.79, *p* = 0.000, *p*_Heterogeneity_ = 0.388; IM and PM: 84.1% vs. 87.2%, OR = 0.79, 95% CI: 0.63–0.99, *p* = 0.039, *p*_Heterogeneity_ = 0.443). However, in Africa, there was a significant difference between EM and IM (66.4% vs. 80.2%, OR = 0.46, 95% CI: 0.24–0.90, *p* = 0.023, *p*_Heterogeneity_ = 0.323), but neither between EM and PM (66.4% vs. 83.3%, OR = 0.47, 95% CI: 0.13–1.74, *p* = 0.258, *p*_Heterogeneity_ = 0.466) nor between IM and PM (80.2% vs. 83.3%, OR = 1.27, 95% CI: 0.31–5.25, *p* = 0.743, *p*_Heterogeneity_ = 0.665) as shown in Figure 6; Table 6. Therefore, it showed that effects of CYP2C19 genetic polymorphisms influence on the *H. pylori* cure rates could be of greater clinical implication in the Asian populations.

**TABLE 6:** Efficacy of CYP2C19 polymorphisms on the cure rates of *H. pylori* in different geographical location of patients.

Subgroup	EM vs. IM				EM vs. PM				IM vs. PM			
	N	OR (95%CI); sig	Het	Cure rate	N	OR (95%CI); sig	Het	Cure rate	N	OR (95%CI); sig	Het	Cure rate
Asian	5515	0.75 (0.61–0.91), *p* = 0.003	*p* = 0.043	EM (80.6%);	3451	0.63 (0.49–0.79), *p* = 0.000	*p* = 0.388	EM (80.6%);	4042	0.79 (0.63–0.99), *p* = 0.039	*p* = 0.443	IM (84.1%);
				IM (84.1%)				PM (87.2%)				PM (87.2%)
Europe	350	0.46 (0.24–0.90), *p* = 0.023	*p* = 0.323	EM (66.4%);	247	0.47 (0.13–1.74), *p* = 0.258	*p* = 0.466	EM (66.4%);	139	1.27 (0.31–5.25), *p* = 0.743	*p* = 0.665	IM (80.2%);
				IM (80.2%)				PM (83.3%)				PM (83.3%)

### The efficacy of CYP2C19 polymorphism on the cure rates of *H. pylori* in triple therapy and quadruple therapy

**FIGURE 7:**
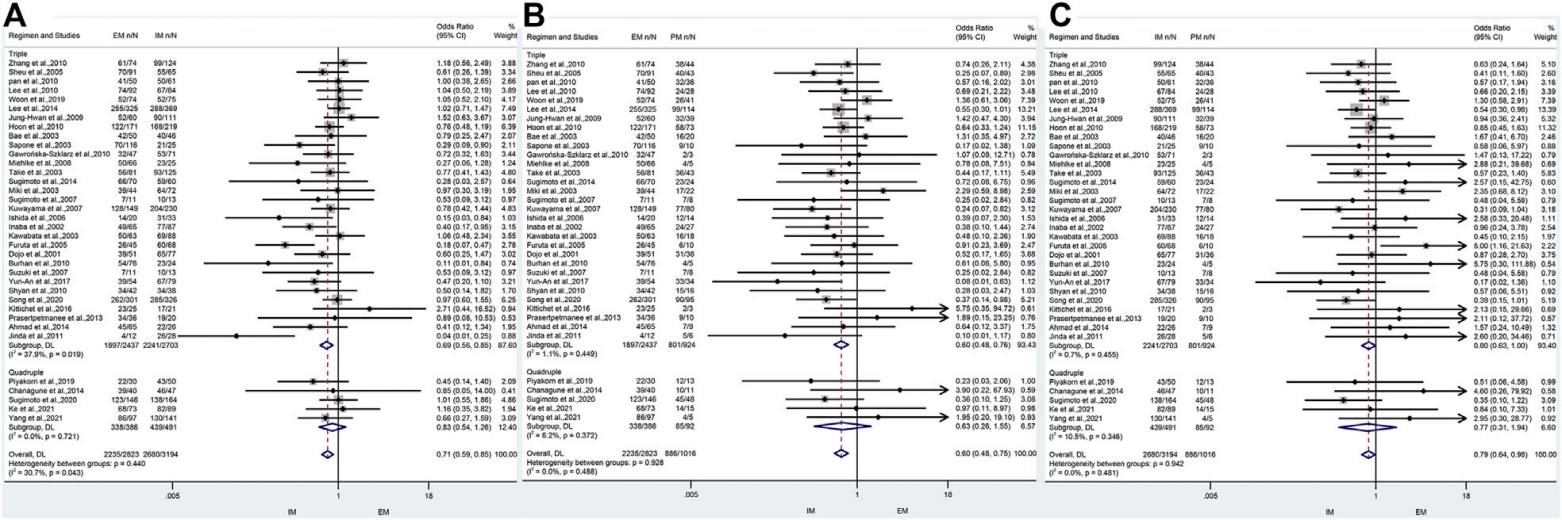
**(A)** Forest plot of EM vs. IM in relation to the *H. pylori* cure rate in the CYP2C19 genotype in different treatment regimens (*p* = 0.043). **(B)** Forest plot of EM vs. PM in relation to the *H. pylori* cure rate in the CYP2C19 genotype in different treatment regimens (*p* = 0.488). **(C)** Forest plot of IM vs. PM in relation to the *H. pylori* cure rate in the CYP2C19 genotype in different treatment regimens (*p* = 0.481). *p* = 0.05, significant heterogeneity or statistically significant, when appropriate. *p* < 0.05, random-effects model; *p* > 0.05, random-effects model. Symbol: 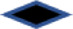, single studies included in the meta-analysis; 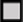, sample size of single studies; 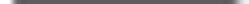, confidence interval (CI); 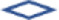, overall pool estimated; 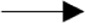, tendency; 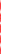, overall pool OR.

Only one study (Ke et al., 2021) about double therapy met the inclusion criteria, but no analysis could be performed for a single study and thus it was excluded. There was a significant difference in *H. pylori* cure rates among all genotypes for triple therapy (EM vs. IM: 77.8% vs. 82.9%, OR = 0.69, 95% CI: 0.56–0.85, *p* = 0.000, *p*_Heterogeneity_ = 0.019; EM vs. PM: 77.8% vs. 86.7%, OR = 0.60, 95% CI: 0.48–0.76, *p* = 0.000, *p*_Heterogeneity_ = 0.449; IM vs. PM: 82.9% vs. 86.7%, OR = 0.80, 95% CI: 0.63–1.00, *p* = 0.050, *p*_Heterogeneity_ = 0.455). On the contrary, there was no significant difference in *H. pylori* cure rates among all genotypes for quadruple therapy (EM vs. IM: 87.6% vs. 89.4%, OR = 0.83, 95% CI: 0.54–1.26, *p* = 0.382, *p*_Heterogeneity_ = 0.440; EM vs. PM: 87.6% vs. 92.4%, OR = 0.63, 95% CI: 0.26–1.55, *p* = 0.312, *p*_Heterogeneity_ = 0.928; IM vs. PM: 89.4% vs. 92.4%, OR = 0.77, 95% CI: 0.31–1.94, *p* = 0.578, *p*_Heterogeneity_ = 0.346) as shown in Figure 7; Table 7. Moreover, overall *H. pylori* cure rates were better with quadruple therapy than those with triple therapy (EM: 87.6% vs. 77.8%, IM: 89.4% vs. 82.9%; PM: 92.4% vs. 86.7%). This might indicate that the cure rate of *H. pylori* with bismuth-containing quadruple therapy is less influenced by the CYP2C19 genotype and a desirable cure rate can be achieved.

**TABLE 7:** Efficacy of CYP2C19 polymorphism on the cure rates of *H. pylori* in triple therapy and quadruple therapy.

Subgroup	EM vs. IM				EM vs. PM				IM vs. PM			
	N	OR (95%CI); sig	Het	Eradication rate	N	OR (95%CI); sig	Het	Eradication rate	N	OR (95%CI); sig	Het	Eradication rate
Triple	5140	0.69 (0.56–0.85), *p* = 0.000	*p* = 0.019	EM (77.8%);	3361	0.60 (0.48–0.76), *p* = 0.000	*p* = 0.449	EM (77.8%);	3627	0.80 (0.63–1.00), *p* = 0.050	*p* = 0.455	IM (82.9%);
				IM (82.9%)				PM (86.7%)				PM (86.7%)
Quadruple	877	0.83 (0.54–1.26), *p* = 0.382	*p* = 0.440	EM (87.6%);	478	0.63 (0.26–1.55), *p* = 0.312	*p* = 0.928	EM (87.6%);	583	0.77 (0.31–1.94), *p* = 0.578	*p* = 0.346	IM (89.4%);
				IM (89.4%)				PM (92.4%)				PM (92.4%)

### Heterogeneity and sensitivity analysis of overall

**FIGURE 8:**
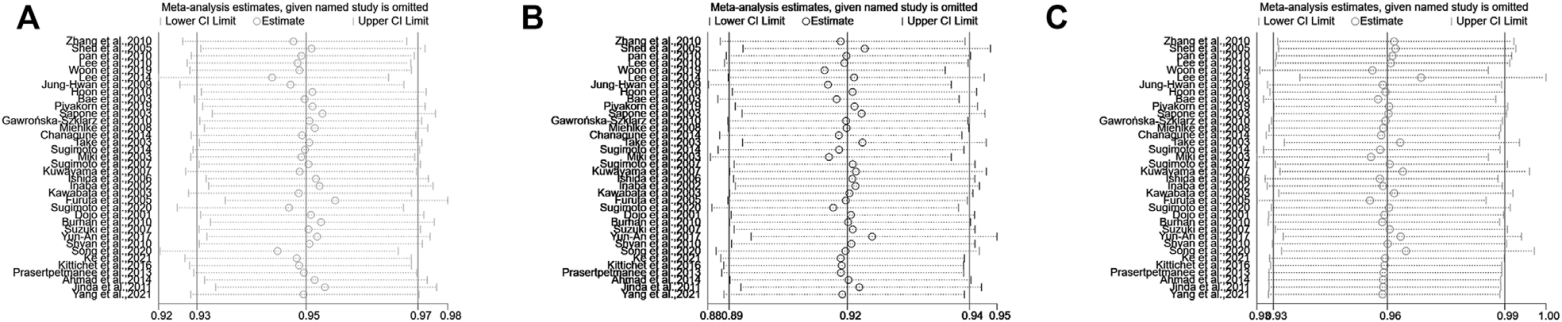
**(A)** Sensitivity analysis of EM vs. IM in relation to the *H. pylori* cure rate in the CYP2C19 genotype. **(B)** Sensitivity analysis of EM vs. PM in relation to the *H. pylori* cure rate in the CYP2C19 genotype. **(C)** Sensitivity analysis of IM vs. PM in relation to the *H. pylori* cure rate in the CYP2C19 genotype.

For the meta-analysis comparing EM vs. IM in relation to the *H. pylori* cure rate in the CYP2C19 genotype, there was a significant heterogeneity across the enrolled studies (*p*_Heterogeneity_ = 0.035, I^2^ = 32.1%), but neither in EM vs. PM (*p*_Heterogeneity_ = 0.478, I^2^ = 0%) nor in IM vs. PM (*p*_Heterogeneity_ = 0.537, I^2^ = 0%). In addition, to assess the stability of the pooled results, sensitivity analysis was carried out to assess the influence of individual studies and sources of heterogeneity on the overall effects for EM vs. IM through repeating the meta-analysis after sequentially omitting each study. As shown in Figure 8, for EM vs. IM, the heterogeneity analysis suggested that a study by Lee et al. (2014), Furuta et al. (2005), and Song et al. (2020) were the main source of heterogeneity. After deleting these three studies (Furuta et al., 2005; Lee et al., 2014; Song et al., 2020), the results were still not changed (EM vs. IM: OR = 0.71, 95% CI: 0.61–0.83, P_Heterogeneity_ = 0.180, I^2^ = 18.2%).

### Publication bias

**FIGURE 9:**
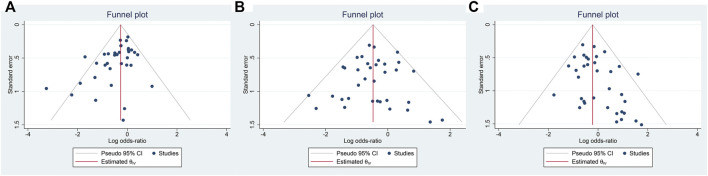
**(A)** Publication bias analysis of EM vs. IM in relation to the *H. pylori* cure rate in the CYP2C19 genotype (t = −3.15, *p* = 0.003). **(B)** Sensitivity analysis of EM vs. PM in relation to the *H. pylori* cure rate in the CYP2C19 genotype (t = −0.34, *p* = 0.737). **(C)** Sensitivity analysis of IM vs. PM in relation to the *H. pylori* cure rate in the CYP2C19 genotype (t = 2.19, *p* = 0.035). Publication bias, *p* = 0.05.

**FIGURE 10:**
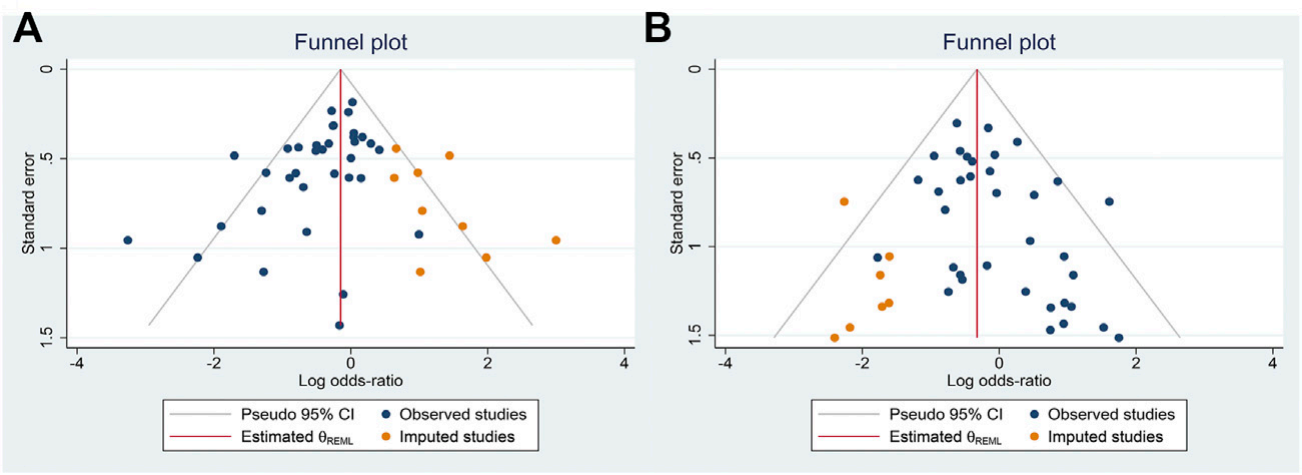
**(A)** Trim-and-fill analysis of EM vs. IM in relation to the *H. pylori* cure rate in the CYP2C19 genotype. Nine studies needed to be filled. **(B)** Trim-and-fill analysis of IM vs. PM in relation to the *H. pylori* cure rate in the CYP2C19 genotype. Seven studies needed to be filled.

To test the publication bias of the included studies, the funnel plot asymmetry test was used. The funnel plots comparing EM vs. PM with *H. pylori* cure rates in the CY2C19 genotype were symmetrical, indicating no publication bias of these studies was presented (EM vs. PM: t = −0.34, *p* = 0.737); however, for EM vs. IM and IM vs. PM, the shape of the funnel plot was obviously asymmetrical and Egger’s test also provided statistical evidence of funnel plot asymmetry (t = −3.15, *p* = 0.003; Figure 8; IM vs. PM: t = 2.19, *p* = 0.035; Figure 9). To adjust these publication biases, a trim-and-fill analysis method (Duval and Tweedie, 2000) was performed. Data demonstrated that the results were stable for EM vs. PM before and after the use of this analysis, but those nine studies and seven studies needed to be filled for EM vs. IM and IM vs. PM, respectively (Figure 10).

## Discussion

In our study, we found that the *H. pylori* cure rate of individuals with the EM genotype was significantly lower than that of those with IM or PM genotypes. Our results also revealed that the CYP2C19 genotype status affected the efficacy of omeprazole-based therapy, but not of pantoprazole- and rabeprazole-based therapy. In addition, a significant difference was observed between CYP2C19 genotypes and *H. pylori* cure rates in therapy of 7-day, but not in the 14-day therapy regimen. In Asia, *H. pylori* cure rates were influenced by the CYP2C19 genotype. But in Europe, whether *H. pylori* cure rates could be influenced by the CYP2C19 genotype would need to be explored in detail with an expanded sample size. Finally, we also found significant differences in *H. pylori* cure rates among the three genotypes in triple therapy, but no significant differences in *H. pylori* cure rates were observed among the three genotypes in quadruple therapy.

To date, the published data showed inconsistent results on the effects of the CYP2C19 genotype on the cure rates of *H. pylori* infections. Our pooled result showed that the CYP2C19 genotype could affect the cure rate of *H. pylori*, and the cure rate of EM genotype was significantly lower than that of the IM or PM genotype, consistent with the results reported by Ormeci et al. (2016) and Fu et al. (2021). Actually, the relative enzyme activity of the EM was roughly 2-fold higher than that of the IM (0.15 vs. 0.08, respectively), which in turn was double as that of the PM (0.08 vs. 0.04) (Kuo et al., 2014), indicating a gene-dosage effect. Individuals with an EM phenotype clear PPIs at a higher rate (Franciosi et al., 2018). However, there was no significant difference in the cure rate of *H. pylori* between the IM genotype and PM genotype in our study.

Clinical research studies have demonstrated that the use of PPIs is the determinant in enhancing the cure rate of *H. pylori* (Kim et al., 2019). PPIs are primarily inactivated in the liver by the microsomal enzyme CYP2C19, and genetic variation in the CYP2C19 gene determines enzyme activity (Zamani et al., 2018). However, some studies provided conflicting evidence for the impact of PPI-related CYP2C19 genetic polymorphisms on PPI-treated cure rates of *H. pylori*. For example, Shah et al. (2021) categorized omeprazole, pantoprazole, and lansoprazole as PPIs that were predominantly metabolized by CYP2C19, but esomeprazole and rabeprazole are minimally or not metabolized by CYP2C19. McNicholl et al. (2012) observed that the clinical efficacy of some new-generation PPIs (esomeprazole and rabeprazole) was not affected by CYP2C19 genetic polymorphisms and that there were higher cure rates for the use of the first-generation PPIs (omeprazole, lansoprazole, and pantoprazole) in EM patients. Padol et al. (2006) indicated that the *H. pylori* cure rate was not affected by CYP2C19 genetic polymorphisms when using lansoprazole or rabeprazole. Moreover, Arenas et al. (2019) described phenotypes of CYP2C19 genetic polymorphisms that did not affect the *H. pylori* cure rate. These controversies could be explained by none of these studies taking into account the effects of antibiotic sensitivity, doses of PPIs, geographic differences, patient compliance, and more. In fact, most of the studies that supported the association between CYP2C19 genetic polymorphisms and *H. pylori* cure rates were based on Asian populations, with a greater proportion of the PM genotype (Arenas et al., 2019). Our meta-analysis showed that the cure rate of *H. pylori* of PPIs, such as omeprazole was effected by CYP2C19 genotypes, consistent with previous reports among the main PPIs, the various influences of the CYP2C19 polymorphisms on metabolic parameters were different (omeprazole > lansoprazole > pantoprazole > rabeprazole) (Zhang et al., 2020). However, in our study, we observed that there was no significant difference in the cure rates between all genotypes with either pantoprazole- or rabeprazole-based double, triple, or quadruple therapies. Rabeprazole has been reported to be mainly metabolized (approximately 85%) *via* a non-enzymatic pathway to thioether-rabeprazole, with only minor involvement of CYP2C19 and CYP3A4 (Hu et al., 2005; Wang et al., 2011), consistent with the results derived from our meta-analysis, suggesting that rabeprazole-based triple or quadruple therapies can be used to eradicate *H. pylori* infection for all patients, with no need in considering the status of CYP2C19 genetic polymorphisms. However, pantoprazole is mainly metabolized by different metabolic enzymes (CYP2C19 and CYP3A4) (Zhang et al., 2020), inconsistent with our results, and the explanations for the discrepancy may be the small numbers of studies (Jung-Hwan et al., 2009; Gawrońska-Szklarz et al., 2010; Lee et al., 2014) and subjects (602 of 5496) included in the meta-analysis. Therefore, rabeprazole and pantoprazole treatment programs were superior to omeprazole in the case of ignoring other influencing factors, such as side effects, cost of treatment, other metabolizer genes affecting other drugs, and more. Moreover, the positioning of potassium-competitive acid blockers (P-CABs) is also worth considering, particularly because vonoprazan, which is not currently approved outside certain Asia-Pacific and South/Central American countries/regions and is actively being investigated for the use in the United States and Europe for conditions necessitating gastric acid suppression (Shah et al., 2021). Vonoprazan is metabolized mainly by CYP3A4/5 and partially by CYP2B6, CYP2C19, and CYP2D6, and its pharmacokinetics and pharmacodynamics may be influenced by genetic variations in the respective genes (Sugimoto et al., 2020). However, our study showed a significant difference only in EM vs. IM in lansoprazole-based therapy, but not in EM vs. PM or IM vs. PM, a significant difference was observed only in EM vs. PM in esomeprazole-based therapy, but not in EM vs. IM or IM vs. PM. Previous reports suggested that the efficacy of lansoprazole is dependent on the CYP2C19 gene status (Fu et al., 2021), but Padol et al. (2006) showed that *H. pylori* therapies with omeprazole are dependent on the CYP2C19 genotype while therapies with lansoprazole and rabeprazole are not. Thus, the conclusion drawn is ambiguous and requires further research. Meanwhile, the articles used in this study were based on the difference between PPI dosages and antibiotic susceptibility, which may affect the conclusion of this study. Therefore, the choice of different PPIs and/or doses should be individualized based on the pharmacogenetics background of each patient.

In addition, we reported for the first time a subgroup analysis regarding treatment duration (7 versus 14 days) and revealed a significantly different cure rate of *H. pylori* for CYP2C19 genotypes (EM vs. PM) in those studies of 7 days, but not of 14 days, indicating that effects of the CYP2C19 genotype on the cure rate of *H. pylori* could be reduced with prolonging of treatment time. The cure rate of *H. pylori* was higher in 14-day therapy than that in 7-day therapy (88.1% vs. 80.8%), Hwang et al. (2015) reported that 14-day therapy was a more effective second-line treatment as compared to the 7-day therapy for *H. pylori* infection in South Korea (Hwang et al., 2015), indicating that the 14-day therapy may prioritize patients with all genotypes, regardless of the effect of other factors. Therefore, the cost effectiveness of a general recommendation of 14-day therapy has to be confirmed by pharmacoeconomic analysis.

Different geographical locations and genetic backgrounds of CYP2C19 could affect the cure rate of *H. pylori*. We collected data about *H. pylori* treatment from several countries and found that the cure rates of the EM genotype and IM genotype were significantly different in Asia, but not between the EM genotype and PM genotype. Therefore, a more detailed subgroup analysis stratified by country or geographical location, including Mainland China, China Taiwan, Japan, South Korea, India, Thailand, and Turkey, should be performed. However, CYP2C19 genetic polymorphism was not associated with the cure rate of *H. pylori* by PPIs-based therapy in Europe. Why are there regional differences? The distribution of PM was reported to show a considerable interethnic variation. For example, East Asian people, including Chinese, South Koreans, and Japanese, have 13%–23% PMs, whereas Caucasians and African-Americans have only less than 6% (Küpfer et al., 1984; Wedlund et al., 1984; Jacqz et al., 1988; Horai et al., 1989; Sanz et al., 1989; Bertilsson et al., 1992; Sohn et al., 1992; Edeki et al., 1996; Kubota et al., 1996), indicating that the frequency of the PM genotype is significantly higher in the Asian population than in those of other ethnic descents (Adachi et al., 2000). In addition, there were differences in dietary habits and economic levels between Asia and Europe, as well as differences in therapeutic schedules and treatment levels. Therefore, it is more clinically relevant to investigate the effect of the CYP2C19 genotype on *H. pylori* treatment in Asia.

Finally, we analyzed the influence of CYP2C19 genotypes on the cure rate of *H. pylori* in patients who received PPI-based triple therapy and quadruple therapies. We found that *H. pylori* treatment by triple therapy was influenced by the CYP2C19 genotype, but quadruple therapy was not. In addition, Fu et al. (2021) also showed that the quadruple therapy was not affected by CYP2C19 genetic polymorphism, consistent with our results. This could be interpreted as bismuth is mainly metabolized by the kidney, and its inhibitory effect on *H. pylori* is mainly through the inhibition of proteases, urokinase, and phospholipase produced by *H. pylori*, all of which are not affected by CYP2C19. Therefore, bismuth-containing quadruple regimens and PPIs (e.g., rabeprazole, esomeprazole, and pantoprazole) are less affected by CYP2C19 genetic polymorphisms and may be more appropriate solutions when only the effects of single genetic polymorphisms are considered (Fu et al., 2021).

Although the effect of the CYP2C19 genotype on the cure rate of *H. pylori* had been reported, our analysis was more comprehensive and the data more detailed than other meta-analyses. Tang et al. (2013) showed that the treatment of *H. pylori* by omeprazole and lansoprazole were affected by CYP2C19 genetic polymorphisms, while esomeprazole and rabeprazole were not affected, consistent with our results, but Tang et al. did not explore whether *H. pylori* treatment by pantoprazole was affected by CYP2C19 genetic polymorphisms due to the inclusion of less data on pantoprazole, and only triple therapy was included, so subgroup analysis of treatment regimens could not be performed, furthermore, subgroup analysis of geography and treatment duration were also not conducted. Recently, Fu et al. (2021) considered the effect of geographical factors on the CYP2C19 genotype, demonstrated the *H. pylori* cure rates of Mainland Chinese were influenced by CYP2C19 genetic polymorphism, whereas China Taiwanese was not affected, they attributed it to possible difference in dietary habits and treatment protocols.

In addition, to investigate whether there were crossover patients in the clinical trial studies, we pooled the country, institution, time, number of patients, and type of the study for each study and found that each study was conducted at a different institution and at a different time, so there were no crossover patients (Table 8).

**TABLE 8:** Sources of included studies.

Reference	Institution	Country	Time	Number	Type of study
Zhang et al. (2010)	The First Affiliated Hospital of Anhui Medical University	China	From June 2006 to January 2008	240	CT
Sheu et al. (2005)	National Cheng Kung University	China	From January 2002 to May 2004	200	CT
Pan et al. (2010)	The First Affiliated Hospital of Nanjing Medical University	China	From May 2008 and January 2009	184	CT
Lee et al. (2010)	The Alice Ho Miu Ling Nethersole Hospital (AHNH), The Chinese University of Hong Kong	China	From June 2004 to December 2005	204	CT
Woon et al. (2019)	Kyung Hee University Hospital, Seoul, Korea.	South Korea	From September 2014 to August 2015	190	CT
Lee et al. (2014)	Seoul National University Bundang Hospital in Korea	South Korea	Between March 2003 and May 2013	2202	CT
Jung-Hwan et al. (2009)	Kangnam St. Mary’s hospital	South Korea	From January 2003 to December 2004	210	CT
Hoon et al. (2010)	Asan Medical Center	South Korea	From May 2006 to September 2008	463	CT
Bae et al. (2003)	Bundang Jesaeng General Hospital from August	South Korea	From 2002 to May, 2003	116	CT
Piyakorn et al. (2019)	Thammasat University	Thailand	NA	100	RCT
Sapone et al. (2003)	St. Orsola Hospital	Italy	Between December, 2000 and December, 2001	143	CS
Gawrońska-Szklarz et al. (2010)	The Pomeranian Medical University, Szczecin, Poland	Poland	NA	139	CT
Miehlke et al. (2008)	The central Department of Medical Microbiology	Germany	NA	103	CT
Chanagune et al. (2014)	Thammasat University Hospital	Thailand	Between December 2012 and December 2013	50	RCT
Take et al. (2003)	Nippon Kokan Fukuyama Hospital	Japan	From June 1998, to January 2001	249	CT
Sugimoto et al. (2014)	The University Hospital of Hamamatsu University School of Medicine	Japan	From September 2009 to March 2013	153	CT
Miki et al. (2003)	Kobe University School of Medicine, Kobe, Japan	Japan	NA	145	CT
Sugimoto et al. (2007)	The University Hospital of Hamamatsu University School of Medicine	Japan	From January 2004 to December 2006	32	CT
Kuwayama et al. (2007)	Dokkyo University School of Medicine, Koshigaya	Japan	NA	459	RCT
Ishida et al. (2006)	Daiko Medical Center, Nagoya University, Nagoya, Japan	Japan	Between July 2004 and October 2005	210	CT
Inaba et al. (2002)	Kagawa Prefectural Central Hospital	Japan	From March 1998 to April 2000	183	CT
Kawabata et al. (2003)	Kyoto Second Red Cross Hospital, Kyoto, Japan	Japan	Between April and October 2000	187	CT
Furuta et al. (2005)	First Department of Medicine, Hamamatsu University School of Medicine, Hamamatsu	Japan	NA	141	CS
Sugimoto et al. (2020)	The Shiga University of Medical Science Hospital	Japan	From April 2015 to December 2019	307	CT
Dojo et al. (2001)	The Second Department of Internal Medicine, Fukui Medical University to	Japan	NA	170	CT
Burhan et al. (2010)	Çukurova University Balcal› Hospital, Department of Gastroenterology	Turkey	Between September 2005 and December 2008	105	CT
Suzuki et al. (2007)	Division of Epidemiology and Prevention, Aichi Cancer Center Research Institute, Japan	Japan	From March to December 1999	142	CT
Yun-An et al. (2017)	Guangzhou First Municipal People’s Hospital	China	From May to December 2014	160	CT
Shyan et al. (2010)	Department of Internal Medicine, Lin Shin Hospital, Taichung	China	Between January 2002 and December 2006	95	CT
Song et al. (2020)	Peking University Third Hospital, Beijing, China	China	Between October 2017 and November 2018	380	RCT
Ke et al. (2021)	Nanfang Hospital of Southern Medical University	China	From November 2015 to October 2019	207	RCT
Piyakorn et al. (2019)	Kyung Hee University Hospital, Seoul, Korea.	Thailand	From September 2014 to August 2015	100	CT
Kittichet et al. (2016)	Thammasat University Hospital, Pathumthani	Thailand	During March 2015 and January 2016	50	RCT
Prasertpetmanee et al. (2013)	Thammasat University Hospital, Thailand	Thailand	Between December 2010 and December 2011	110	RCT
Ahmad et al. (2014)	Mansoura University Pediatric Hospital	Egyptian	Between September 2011 and December 2012.	100	CT
Jinda et al. (2011)	The Mie University Hospital, Doshinkai Toyama Hospital, Nagai Hospital or Saiseikai Matsusaka General Hospital	Japan	NA	45	CT

Abbreviations: CT, clinical trial; CS, comparative study; NA, not available; RCT, randomized controlled trial.

This study may have the following limitations. First, the sample size of the included studies was not uniform, and the between-study difference may be large. Some studies had a small sample size (at least 32 cases) and the largest sample size (at most 2202) with a 68-fold difference in between. Second, there were limitations of the included literature. In this study, reviewed English scientific journals were included and other language literature works were excluded, which may cause a certain bias to the research results. Third, there was only published literature included. There may still be some documents with negative results that have not been published and included in the analysis, which would also affect the results of this study. Fourth, documents with incomplete data are excluded, which may lead to selection bias. Finally, the effects of dose of PPIs, antibiotic sensitivity, patient compliance, and other genotypes (IL-1β and CYP3A4) on *H. pylori* cure rates were not considered.

Taken together, our study concluded that there is a significant difference in the cure rate of *H. pylori* between EM and PM/IM genotypes, especially for the treatment with omeprazole, 7-day therapy, and triple therapy. Therefore, to overcome or minimize the effect of the CYP2C19 genotype on *H. pylori* cure rates, the appropriate PPIs and treatment plan should be selected according to the genotypes of CYP2C19.

## Data Availability

The original contributions presented in the study are included in the article/Supplementary Material; further inquiries can be directed to the corresponding author.
